# Metabolic Engineering of Crocin Biosynthesis in *Nicotiana* Species

**DOI:** 10.3389/fpls.2022.861140

**Published:** 2022-03-08

**Authors:** Oussama Ahrazem, Changfu Zhu, Xin Huang, Angela Rubio-Moraga, Teresa Capell, Paul Christou, Lourdes Gómez-Gómez

**Affiliations:** ^1^Departamento de Ciencia y Tecnología Agroforestal y Genética, Instituto Botánico, Universidad de Castilla-La Mancha, Campus Universitario, Albacete, Spain; ^2^Department of Plant Production and Forestry Science, University of Lleida-Agrotecnio Centre de Recerca en Agrotecnologia (CERCA) Center, Lleida, Spain; ^3^School of Life Sciences, Changchun Normal University, Changchun, China; ^4^Catalan Institute for Research and Advanced Studies (ICREA), Catalan Institute for Research and Advanced Studies, Barcelona, Spain

**Keywords:** apocarotenoids, biotechnology, CsCCD2L, crocins, *Nicotiana*, saffron

## Abstract

Crocins are high-value soluble pigments that are used as colorants and supplements, their presence in nature is extremely limited and, consequently, the high cost of these metabolites hinders their use by other sectors, such as the pharmaceutical and cosmetic industries. The carotenoid cleavage dioxygenase 2L (*CsCCD2L*) is the key enzyme in the biosynthetic pathway of crocins in *Crocus sativus*. In this study, *CsCCD2L* was introduced into *Nicotiana tabacum* and *Nicotiana glauca* for the production of crocins. In addition, a chimeric construct containing the *Brevundimonas* sp. β-carotene hydroxylase (*BrCrtZ*), the *Arabidopsis thaliana ORANGE* mutant gene (*AtOrMut*), and *CsCCD2L* was also introduced into *N. tabacum*. Quantitative and qualitative studies on carotenoids and apocarotenoids in the transgenic plants expressing *CsCCD2L* alone showed higher crocin level accumulation in *N. glauca* transgenic plants, reaching almost 400 μg/g DW in leaves, while in *N. tabacum* 36 μg/g DW was obtained. In contrast, *N. tabacum* plants coexpressing *CsCCD2L*, *BrCrtZ*, and *AtOrMut* accumulated, 3.5-fold compared to *N. tabacum* plants only expressing *CsCCD2L*. Crocins with three and four sugar molecules were the main molecular species in both host systems. Our results demonstrate that the production of saffron apocarotenoids is feasible in engineered *Nicotiana* species and establishes a basis for the development of strategies that may ultimately lead to the commercial exploitation of these valuable pigments for multiple applications.

## Introduction

Carotenoids are an important group of natural and lipid-soluble pigments which humans cannot synthesize, thus acquiring them through diet. In humans, these molecules play multiple physiological and nutritional functions (Fiedor and Burda, 2014). Carotenoids are synthesized by plants, algae, fungi, and bacteria, displaying a yellow to red coloration, depending on the type of carotenoid and the concentration they reach in the different cells. Carotenoids are widely used as food colorants, nutraceuticals, animal feed additives, cosmetic ingredients, and health supplements (Fraser and Bramley, 2004). In all living organisms, carotenoids act as substrates to produce apocarotenoids (Ahrazem et al., 2016a). These metabolites are not simply breakdown products of carotenoids, as their functions include those of signaling and hormonal (Walter et al., 2010; Eroglu and Harrison, 2013; Jia et al., 2017). Apocarotenoids are present as volatile and soluble compounds. Crocins are water-soluble pigments of high commercial value, used mostly in food and to a lesser extent, in the pharmaceutical industries (Ahrazem et al., 2015). Crocins are glycosylated derivatives of the apocarotenoid crocetin and they exhibit a strong coloring capacity. In addition, crocins are powerful free radical quenchers, which is associated with the broad range of their health benefits (Nam et al., 2010; Georgiadou et al., 2012; Christodoulou et al., 2015; Bukhari et al., 2018). Interest in the therapeutic properties of crocins is increasing due to their analgesic and sedative properties (Amin and Hosseinzadeh, 2012), neurological protection, and anticancer activities (Finley and Gao, 2017; Skladnev and Johnstone, 2017). Furthermore, clinical trials indicate that crocins have a positive effect in the treatment of depression and dementia (Lopresti and Drummond, 2014; Mazidi et al., 2016). However, commercial utilization of crocins from saffron is limited due to elevated prices, a consequence of the high labor costs in harvesting and processing flower material (Ahrazem et al., 2015).

*Crocus sativus* is the main natural source of crocins, which accumulate at high levels during the development of the stigma and confer a dark red coloration (Moraga et al., 2009). Together with the safranal (2,6,6-trimethyl-1,3-cyclohexadiene-1-carboxaldehyde), the precursor of picrocrocin (β-D-glucopyranoside of hydroxyl-β-cyclocitral) is responsible for the flavor of the saffron spice (Tarantilis et al., 1995). In addition to *Crocus*, gardenia (*Gardenia jasminoides*) fruits are also a commercial source of crocins, but on a much lower scale, as they do not accumulate picrocrocin (Pfister et al., 1996; Moras et al., 2018). Among other plants that produce crocins, but which are not commercially exploited due to the low quantities accumulated, are *Buddleja* species (Liao et al., 1999). Therefore, only a few plant species can synthesize crocins, and this is because the carotenoid cleavage dioxygenases (CCDs) responsible for the production of crocins are not usually present in plants.

**FIGURE 1:**
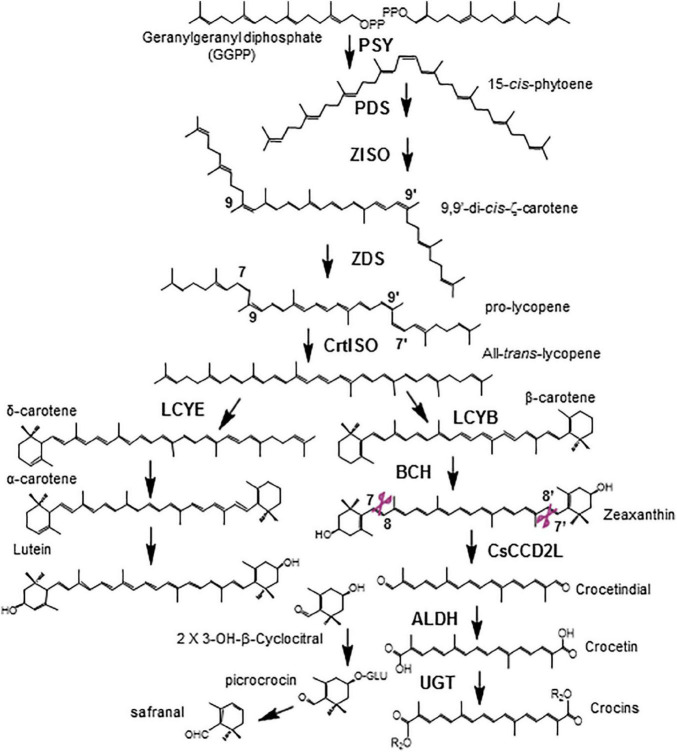
Biosynthetic pathway of crocins in saffron. PSY, phytoene synthase; PDS, phytoene desaturase; Z-ISO, ζ-carotene isomerase; ZDS, ζ-carotene desaturase; CrtISO, carotene isomerase; LCYB, β-lycopene cyclase; LCYE, ε-lycopene cyclase; BCH, β-carotene hydroxylase; ALDH, aldehyde dehydrogenase. 3-OH-β-cyclocitral also known as HTCC (hydroxy-2,6,6-trimethyl-1-cyclohexen-1-carboxaldehyde). Cleavage positions recognized by CsCCD2L are shown in purple.

In *Crocus*, *Gardenia*, and *Buddleja* species, zeaxanthin is the precursor of crocetin (Figure 1). Cleavage of the zeaxanthin molecule at the 7,8 and 7′,8′ double bonds generates one molecule of crocetin dialdehyde and two molecules of 4-hydroxy-2,6,6-trimethyl-1-cyclohexene-1-carboxaldehyde (HTCC) (Frusciante et al., 2014; Ahrazem et al., 2016c,^2017^). Crocetin dialdehyde is further converted to crocetin by the action of aldehyde dehydrogenase enzymes (ALDH), and crocetin is the substrate of glucosyltransferases (UGTs) that catalyze the formation of crocins, catalyzing the transfer of glucose molecules to both ends of the crocetin molecule (Moraga et al., 2004; Nagatoshi et al., 2012; Diretto et al., 2021). The HTCC molecule is also recognized by UGTs, resulting in the formation of picrocrocin, which is further metabolized to render safranal (Figure 1; López et al., 2021). In *C. sativus* the CCD2 enzyme catalyzed the cleavage of zeaxanthin at 7,8;7′,8′ double bonds (Frusciante et al., 2014). CsCCD2L is a plastidic enzyme that belongs to a new CCD subfamily only isolated from *Crocus* species (Ahrazem et al., 2016c), which is closely related to the CCD1 subfamily (Ahrazem et al., 2016a,b). In *Buddleja davidii*, and *G. jasminoides*, the CCD enzymes that catalyzed the same reaction belong to the CCD4 subfamily of CCDs (Ahrazem et al., 2016a; Xu et al., 2020). The *Buddleja* enzymes, BdCCD4.1 and BdCCD4.3, are as well plastidic enzymes expressed in flowers (Ahrazem et al., 2017).

The carotenoid biosynthetic pathway has been modified by genetic engineering in higher plants to increase the general amount of carotenoids and/or to produce specific carotenoids (Farre et al., 2014). In previous studies, *CsCCD2L* was transiently expressed alone and was sufficient to allow the significant crocins accumulation in tobacco plants (Diretto et al., 2019; Marti et al., 2020; López et al., 2021). In this study, we achieved a stable expression of the *CsCCD2L* gene alone or in combination with genes involved in the carotenoid biosynthesis and accumulation, like *BrCrtZ* and *AtOrMut*, to increase the availability of substrates for CsCCD2L activity. We chose the *Nicotiana* species as a heterologous host. The high metabolic versatility, non-food status, and high biomass production of the genus *Nicotiana* make this genus one of the most promising biofactories for the sustainable production of high-value metabolites (Molina-Hidalgo et al., 2021). Metabolic analyses showed the accumulation of these nutritional substances reaching in *Nicotiana glauca* almost 400 μg/g DW, which indicates that *N. glauca* is a better system than *Nicotiana tabacum* to produce crocins because the transgenic *N. glauca* plants expressing CsCCD2L alone could accumulate much higher amounts of crocins as compared with the transgenic *N. tabacum* plants coexpressing *CsCCD2L*, *BrCrtZ*, and *AtOrMut* genes.

## Materials and Methods

### Plant Material

Wild-type (Wt) tobacco (*N. glauca* and *N. tabacum* cv. SR1) and transgenic plants were grown in pots with soil (Traysubstrat, Klasmann-Deilmann GmbH, Postfach 1250, 49741 Geeste, Germany) in a controlled growth chamber with a 25/20°C day/night temperature regime, a 12-h photoperiod (mean irradiance 100 μmol m^–2^ s^–1^) and 60–90% relative humidity. The fully expanded mature leaves (the 5th and 6th leaves) were collected from five Wt and five transgenic plants for each line. All materials were frozen in liquid nitrogen and stored at −80°C.

After leaf sample collection, Wt and transgenic *N. glauca* plants were grown in a controlled growth chamber with a 25/20°C day/night temperature regime, a 12-h photoperiod (mean irradiance 400 μmol m^–2^ s^–1^) and 60–90% relative humidity. Wt and transgenic tobacco plants were self-pollinated to obtain seeds.

### Vector Construction

Two plasmids were created to evaluate their ability to engineer the saffron apocarotenoid pathway in *N. glauca* and *N. tabacum* (Supplementary Figure 1). The tobacco Ubi.U4, *Arabidopsis* AtUBQ10, and CaMV35S were used to drive the expression of the transgenes. The Goldenbraid strategy was followed to construct the vectors (Sarrion-Perdigones et al., 2013, 2014). Briefly, the complete open reading frames of *CsCCD2L*, *BrCrtZ*, and *AtOrMut* were domesticated by removing *Bsm*BI and *Bsa*I restriction sites in the original sequence using the primers listed in Supplementary Table 1. The products were cloned in the level 0 vector pUPD2 of the Goldenbraid modular cloning system. The resulting plasmids pUPD2-CsCCD2L, pUPD2-BrCrtZ, and pUPD2-AtOrMut were then used to construct 2 recombinant binary vectors as follows: pDGB3Ω1[p35S:CCD2L:T35S-pNos:Hyg:T35S] and pDGB3α1 [p35S:CCD2L:T35S-pNos:Hyg:T35S-pTUBI4U:BrCrtZ:T35S-pA tUBQ10:AtOrMut:T35S].

### Transformation of *Agrobacterium* and Tobacco

*Agrobacterium tumefaciens* strain LBA4404 was used for the above binary plasmid transformation. This strain was grown on YEB medium supplemented with 50 μg/ml rifampicin and 50 μg/ml gentamicin final concentrations for 2 days at 28°C. The plasmids pDGB 3Ω1[p35S:CCD2L:T35S-pNos:Hyg:T35S] and pDGB3α1[p35S :CCD2L:T35S-pNos:Hyg:T35S-pTUB I4U:BrCrtZ:T35S-pAtUBQ10:AtOrMut:T35S] were introduced into the bacteria by electroporation. Clones were selected on YEB agar plates containing 100 μg/ml rifampicin, 50 μg/ml spectinomycin or 50 μg/ml kanamycin, and 25 μg/ml gentamicin.

*Agrobacterium*-mediated transformation of both tobacco species (*N. glauca* and *N. tabacum*) was performed according to the leaf disc method of Horsch et al. (1985). For the selection of transformants, the antibiotic hygromycin B at a final concentration of 50 μg/ml was used.

### Carotenoid and Apocarotenoid Extraction and Quantification

Metabolite extraction and analysis differed depending on the nature of the metabolites. Polar and apolar metabolites were extracted from 50 to 5 mg of lyophilized leaves, respectively. For the analysis of polar metabolites, leaves were extracted in cold 50% methanol. The soluble fractions were analyzed by a high-performance liquid chromatography-diode array detector (HPLC-DAD) as previously described (Marti et al., 2020). The insoluble fractions (carotenoids) were extracted with 1:2 cold extraction solvents (50:50 methanol:CHCl_3_), and analyzed by HPLC-DAD as previously described (Marti et al., 2020). Pigments were quantified by integrating peak areas that were converted to concentrations by comparison with the standards and as reported before (Diretto et al., 2019). All the samples were analyzed in triplicate.

### mRNA Expression

Total RNA was isolated using RNeasy Plant Mini Kit (Qiagen, Germany), after DNase treatment, first-strand cDNA synthesis was performed using the SuperScript First-Strand Synthesis System (Takara, Otsu, Japan) primed with oligo (dT)18 following the manufacturer’s instructions. qRT-PCR was performed with a Fluorescent Quantitative PCR Detector (Apply Biosystems). SYBR Green real-time PCR Master Mix (Promega) was used. The amplified DNA fragments for each gene were confirmed by Sanger sequencing. The actin gene was used as a reference gene. qRT-PCR products were assessed by melting curve and gel electrophoresis to ensure the specificity of the amplification in the reactions. Three technical replicates were carried out for each biological sample. Conditions for qRT-PCR cycling were 95°C for 3 min, 95°C for 20 s, 60°C for 20 s, 40 cycles. The relative expression level of each gene was calculated using the 2^–Δ^ ^Δ^ *^Cq^* method. Primers used in the qRT-PCR analysis are listed in Supplementary Table 1.

## Results

### Introduction of *CsCCD2L* in the *Nicotiana glauca* and *Nicotiana tabacum* Genomes

We set out to produce crocins in two different host tobacco species *N. tabacum* and *N. glauca*. For this purpose, two binary vectors were constructed using the Goldenbraid strategy (Sarrion-Perdigones et al., 2013, 2014). The first construct contained only *CsCCD2L* transgene under the control of the cauliflower mosaic virus (CaMV) 35S promoter together with the hygromycin phosphotransferase selectable marker (*hpt*) gene (Supplementary Figure 1). The second construct carried three genes: *CsCCD2L*, *BrCrtZ*, and *AtOrMut* is driven by the CaMV35S promoter, the tobacco polyubiquitin Ubi.U4, and the *Arabidopsis* AtUBQ10 promoter, respectively, together with the hygromycin gene as a selection marker (Supplementary Figure 1).

**FIGURE 2:**
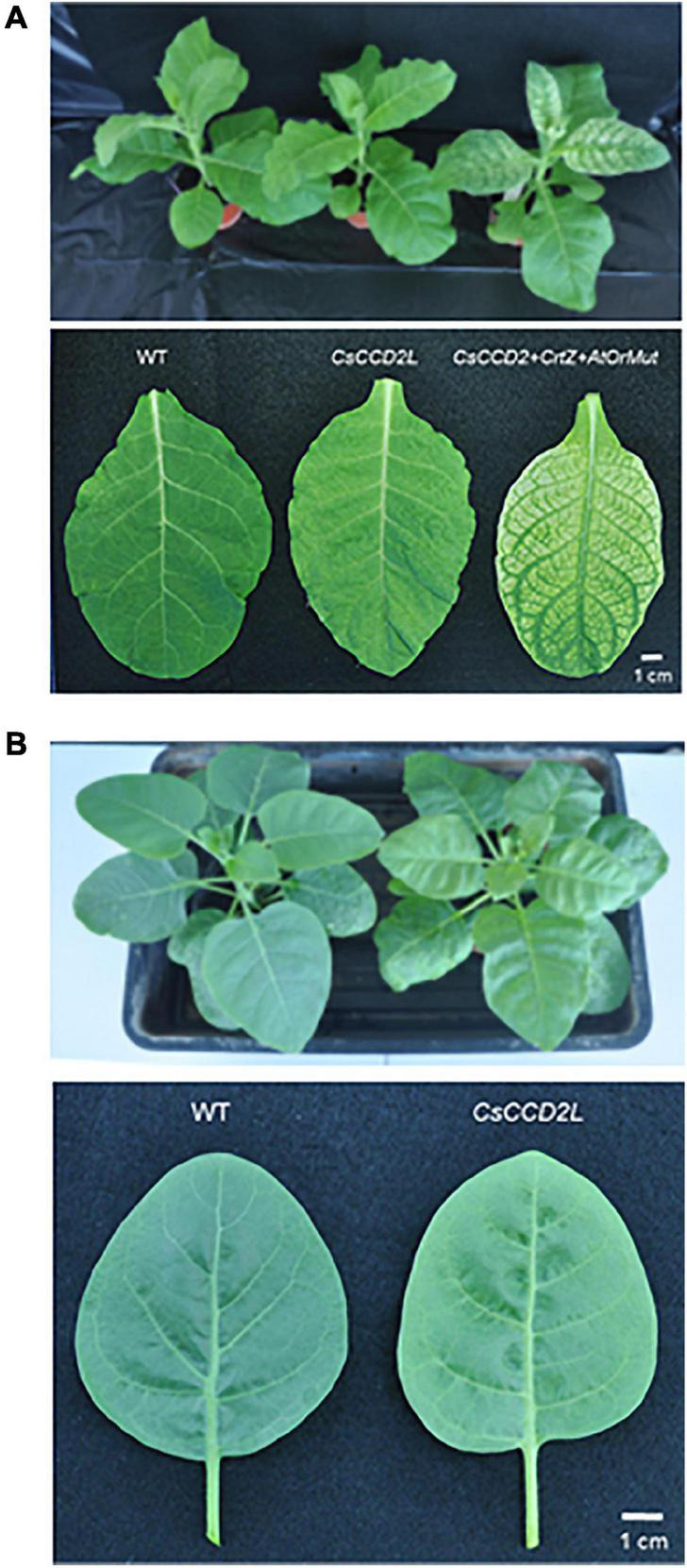
The phenotypes of tobacco plants. **(A)** Plants and sixth leaves from *Nicotiana tabacum* wild-type (Wt) control and T2 homozygous lines expressing *CsCCD2L* or coexpressing *CsCCD2L* + *BrCrtZ* + *AtOrMut* genes. **(B)** Plants and sixth leaves from *N. glauca* Wt control and T1 hygromycin resistant plants expressing *CsCCD2L* gene.

To produce crocins in stably transgenic plants, two tobacco species (*N. tabacum* and *N. glauca*) were transformed with *CsCCD2L* under the control of the CaMV35S promoter (Supplementary Figure 1). Seventy-four putative transgenic *N. tabacum* plants (T0) were obtained. Four out of these seventy-four plants showed a bleaching phenotype (Supplementary Figure 2). All plants were screened for apocarotenoid analysis in green leaves by HPLC, and all these transgenic plants accumulated crocins. Wt and all the crocin accumulating transgenic *N. tabacum* T0 plants were fertile and self-pollinated to obtain T1 seeds. *N. tabacum* T1 seeds were plated on hygromycin B (at a final concentration of 50 μg/ml) selection MS medium. All the lines showed resistance/susceptible segregation under hygromycin selection. T1 and T2 transgenic plants were grown in a greenhouse as described in section “Materials and Methods” (mean irradiance 100 μmol m^–2^ s^–1^) and exhibited a normal phenotype (Figure 2) and were fertile. Based on the segregation patterns of hygromycin-resistant sensitive seedlings, 29 out of the 55 lines were single locus for the introduced transgene. Analysis of genomic DNA confirmed the presence of the intact *CsCCD2L* cassette.

Ten putative transgenic *N. glauca* plants (T0) were obtained and screened by HPLC-DAD analysis. All of these 10 transgenic T0 lines produced crocins in leaves. After leaf sample collection, Wt, and transgenic *N. glauca* T0 plants were grown in a controlled growth chamber with a 25/20°C day/night temperature regime, a 12-h photoperiod (mean irradiance 400 μmol m^–2^ s^–1^) and 60–90% relative humidity. Wt and all the crocin accumulating transgenic *N. glauca* T0 plants were fertile and were self-pollinated to obtain T1 seeds, which took approximately one and a half years in our controlled growth chamber. The transgenic *N. glauca* T1 seeds were plated on hygromycin B (at a final concentration of 50 μg/ml) selection MS medium. All the lines showed resistance/susceptible segregation under hygromycin selection. T1 transgenic plants were grown in a greenhouse as described in section “Materials and Methods” (mean irradiance 100 μmol m^–2^ s^–1^) and exhibited a faint yellow leaf phenotype (Figure 2). Based on the segregation of hygromycin-resistant sensitive seedlings, seven out of the ten lines were deduced to be single locus transgene insertion lines. Analysis of genomic DNA confirmed the presence of the intact *CsCCD2L* cassette similar to *N. tabacum*.

### Introduction of *CsCCD2L, BrCrtZ*, and *AtOrMut* Mutant Genes in the *Nicotiana tabacum* Genome

The flower formation of *N. glauca* depends very much on the season, even in a greenhouse. The plant prefers long-day conditions and high intensity of sunlight. Thus, it may take up to 1 year after seedling transfer to the soil before the first flowers set seeds (Gerjets et al., 2007). Therefore, we have chosen the faster growing *N. tabacum* as a model to improve crocin biosynthesis in leaves by transformation with one binary vector carrying *CsCCD2L*, *BrCrtZ*, and *AtOrMut* mutant genes driven by three different promoters (CaMV35S promoter, *N. tabacum* Ubi.U4 promoter, and *Arabidopsis* ubiquitin 10 promoters, respectively) (Supplementary Figure 1). Seventy-five putative transgenic *N. tabacum* T0 plants were generated and screened by crocin analysis in leaves. Thirty-two transgenic plants accumulated crocins in leaves. All the crocin accumulating transgenic *N. tabacum* T0 plants were fertile and self-pollinated to obtain T1 seeds. T1 seeds were plated on a selection MS medium containing hygromycin B. All 32 lines showed resistance/susceptible segregation for hygromycin selection. T1 and T2 transgenic plants were grown in a greenhouse (mean irradiance 100 μmol m^–2^ s^–1^) and exhibited a yellow leaf phenotype (Figure 2) and were fertile. Based on the segregation of hygromycin-resistant and hygromycin-sensitive tobacco seedlings on selection MS medium supplemented hygromycin B, 15 out of the 32 lines were deduced to be single-locus transgene insertion lines. The genomic DNA was further verified using PCR to check for the presence of *CsCCD2L*, *BrCrtZ*, and *AtOrMut* mutant transgenes.

### Crocin Accumulation in Transgenic *Nicotiana glauca* and *Nicotiana tabacum*

**FIGURE 3:**
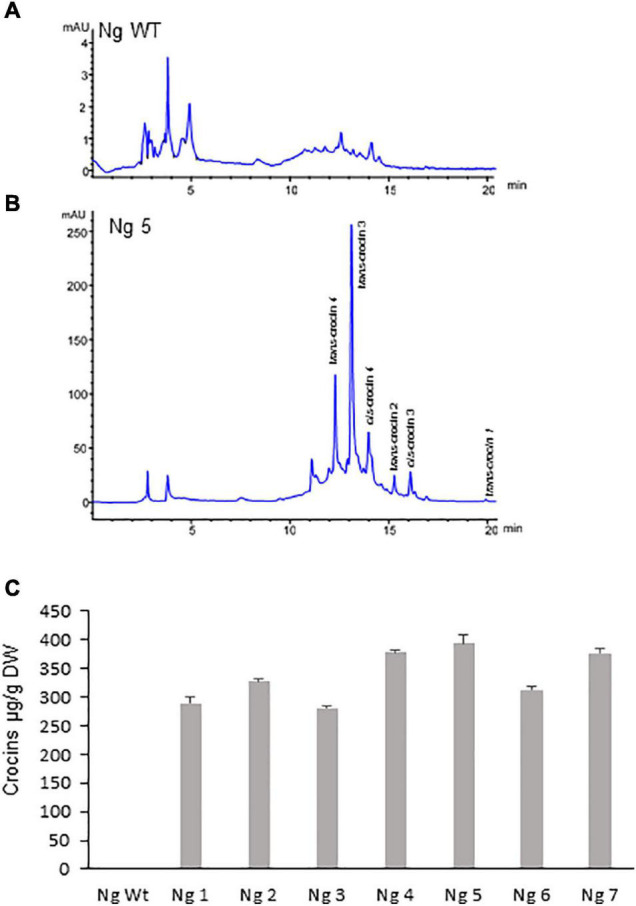
Accumulation of apocarotenoids in Wt and transgenic T1 lines of *Nicotiana glauca* expressing *CsCCD2L*. **(A)** High-performance liquid chromatography-diode array detector (HPLC-DAD) analysis of polar extracts of Wt *N. glauca* leaves at 440 nm. **(B)** HPLC-DAD analysis of polar extracts of transgenic *N. glauca* leaves at 440 nm. Note that peaks for the abundant crocins in the transgenic lines (*trans*-crocin 4, *trans*-crocin 3, *cis*-crocin 4, *trans*-crocin 2, *cis*-crocin 3, and *trans*-crocin 1) are completely absent from Wt plants. mAU, milli-absorbance units. **(C)** Apocarotenoid accumulation in leaves of Wt and transgenic lines. Analyses were done in triplicate. Error bars represent the SD. DW, dry weight.

**FIGURE 4:**
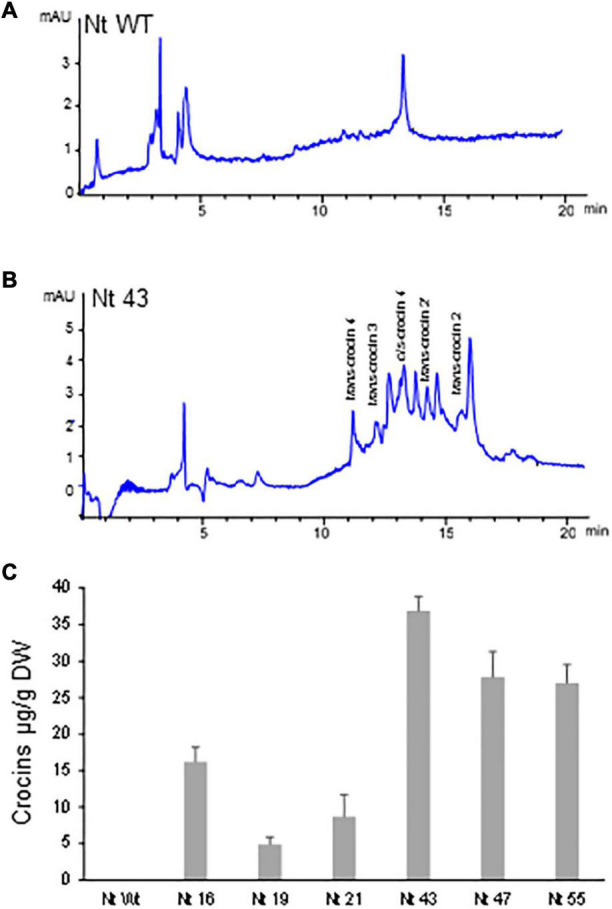
Accumulation of apocarotenoids in wild-type (WT) and transgenic T2 homozygous lines of *Nicotiana tabacum* expressing *CsCCD2L* gene. **(A)** HPLC-DAD analysis of polar extracts of Wt leaves at 440 nm. **(B)** HPLC-DAD analysis of polar extracts of transgenic leaves at 440 nm. Peaks for the abundant crocins in the transgenic lines (*trans*-crocin 4, *trans*-crocin 3, *cis*-crocin 4, *trans*-crocin 2′, and *trans*-crocin 2) are completely absent from Wt plants. mAU, milli-absorbance units. **(C)** Apocarotenoid accumulation in leaves of Wt and transgenic lines. Analyses were done in triplicate. Error bars represent the SD. DW, dry weight.

Polar aqueous extracts from seven T1 *N. glauca* and six T2 *N. tabacum* hygromycin resistant plants were subjected to extraction and analysis for the *de novo* production of crocins. Identification and quantification of the amounts of crocins were conducted by HPLC-DAD using the method described by Diretto et al. (2019). When compared with the chromatogram of the extract of the Wt *N. glauca* (Figures 3A,B) and *N. tabacum* (Figures 4A,B) the chromatograms of the transgenic lines showed several peaks with maximum absorbance from 433 to 440 nm. Further analyses of the retentions times and the spectra data compared with the standards for crocins led to the identification of crocins with different degrees of glycosylation, ranging from one to four glucose molecules (Figures 3B, 4B). Such apocarotenoids were absent in the Wt extracts.

Lines from *N. glauca* accumulated a 10-fold higher amount of crocins than *N. tabacum* lines. Line Ng5 accumulated ca: 400 μg/g DW of crocins (Figures 3C, 4C).

**FIGURE 5:**
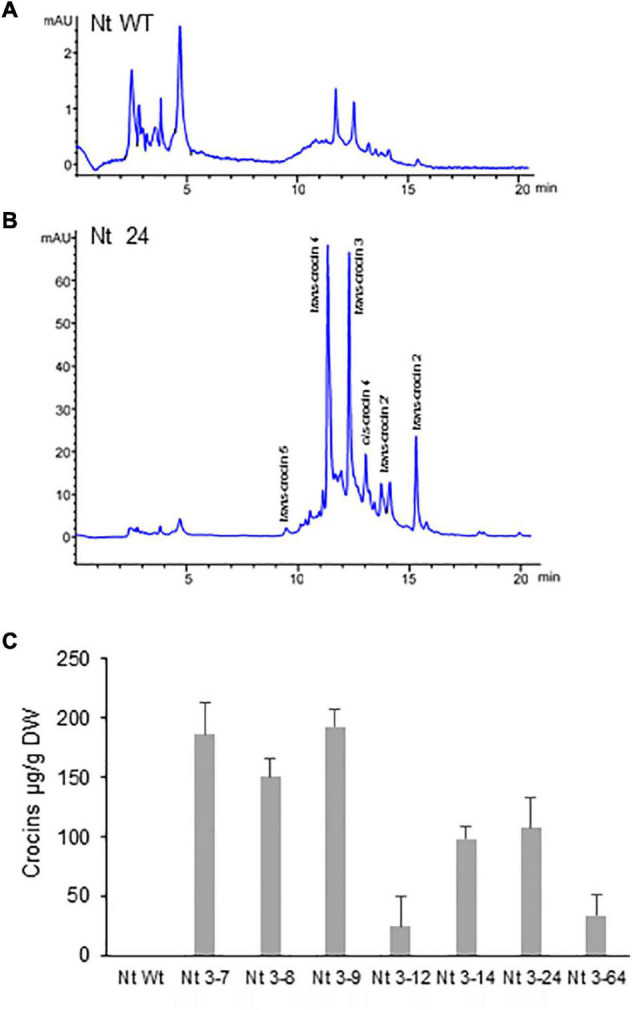
Accumulation of apocarotenoids in wild-type (Wt) and transgenic T2 homozygous lines of *Nicotiana tabacum* coexpressing *CsCCD2L* + *BrCrtZ* + *AtOrMut* genes. **(A)** HPLC-DAD analysis of polar extracts of WT leaves at 440 nm. **(B)** HPLC-DAD analysis of polar extracts of transgenic leaves at 440 nm. Peaks for the abundant crocins in the transgenic lines (*trans*-crocin 4, *trans*-crocin 3, *cis*-crocin 4, *trans*-crocin 2′, and *trans*-crocin 2) are completely absent from Wt plants. mAU, milli-absorbance units. **(C)** Apocarotenoid accumulation in leaves of Wt and transgenic lines. Analyses were done in triplicate. Error bars represent the SD. DW, dry weight.

To evaluate whether the introduction of *AtOrMut* and *BrCrtZ* genes in addition to *CsCCD2L* can boost the level of crocins in *N. tabacum*, polar extracts from seven T2 homozygous lines derived from single-locus transgene insertion lines were analyzed to determine the amount of crocins (Figure 5). All the lines accumulated higher levels of crocins as compared with those lines from *N. tabacum* expressing *CsCCD2L* alone. Interestingly, *N. glauca* transgenic plants accumulated far higher levels of crocins. Line Nt24 was the top line expressing the three transgenes in *N. tabacum*, accumulating crocins to 136 μg/g DW.

### Carotenoid Profiles in Transgenic *Nicotiana glauca* and *Nicotiana tabacum*

**FIGURE 6:**
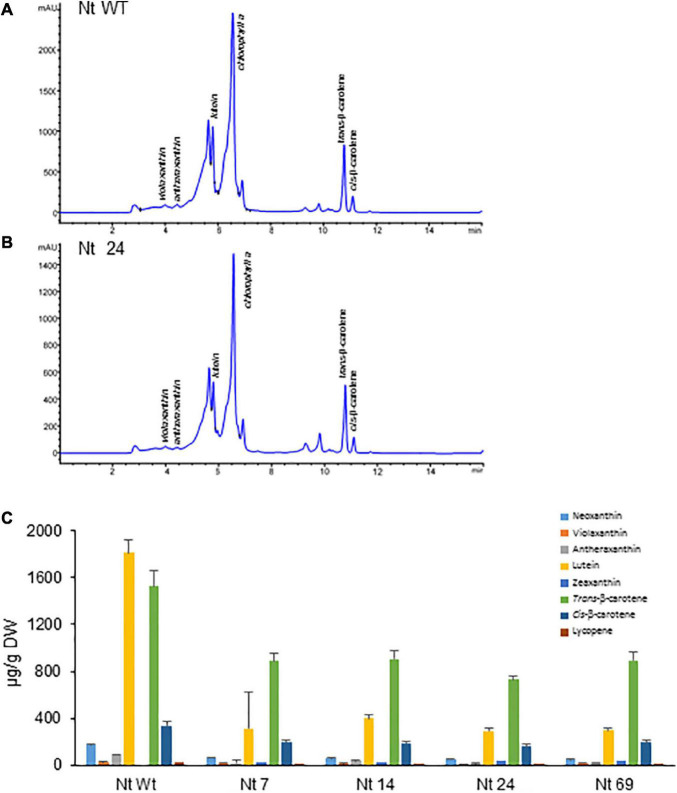
Accumulation of carotenoids in Wt and transgenic T2 lines of *Nicotiana tabacum* expressing *CsCCD2L* + *BrCrtZ* + *AtOrMut* genes. **(A)** HPLC-DAD analysis of apolar extracts of Wt leaves at 450 nm. **(B)** HPLC-DAD analysis of apolar extracts of transgenic leaves at 450 nm. mAU, milli-absorbance units. **(C)** Carotenoid levels in leaves of Wt and transgenic lines. Analyses were done in triplicate. Error bars represent the SD. DW, dry weight.

**FIGURE 7:**
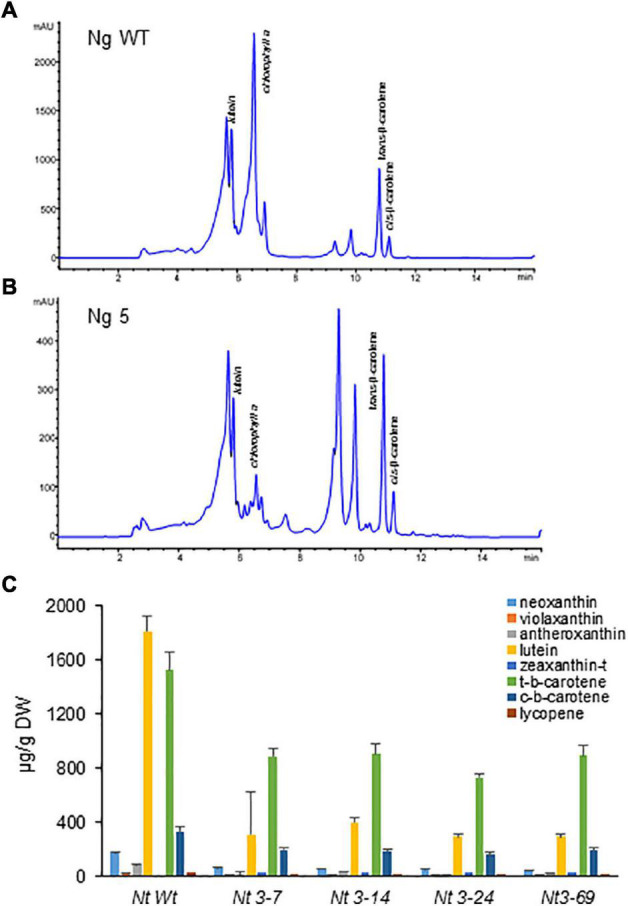
Accumulation of carotenoids in Wt and transgenic T1 lines of *Nicotiana glauca* expressing *CsCCD2L* gene. **(A)** HPLC-DAD analysis of apolar extracts of Wt leaves at 450 nm. **(B)** HPLC-DAD analysis of apolar extracts of transgenic leaves at 450 nm. mAU, milli-absorbance units. **(C)** Carotenoid levels in leaves of Wt and transgenic lines. Analyses were done in triplicate. Error bars represent the SD. DW, dry weight.

Lines accumulating higher levels of crocins were selected for more in-depth analyses. Carotenoids were extracted from four *N. tabacum* lines expressing the three transgenes (*CsCCD2L*, *BrCrtZ*, and *AtOrMut*), three *N. glauca* lines, and Wt controls and analyzed by HPLC-DAD (Figures 6, 7 and Supplementary Tables 2, 3). In general, the carotenoid content of all transgenic lines was much lower compared to their corresponding Wt controls. The most prominent differences in the carotenoid composition of the analyzed leaves were for neoxanthin, β-carotene, and lutein. Neoxanthin, β-carotene, and lutein levels were substantially decreased in the leaves of transgenic plants compared with Wt (Figures 6C, 7C and Supplementary Tables 2, 3). Changes in the levels of chlorophyll were more pronounced in transgenic plants of *N. glauca* than *N. tabacum* (Figures 6B, 7B).

### Expression Analysis of Transgenes and Selected Endogenous Carotenogenic Genes

**FIGURE 8:**
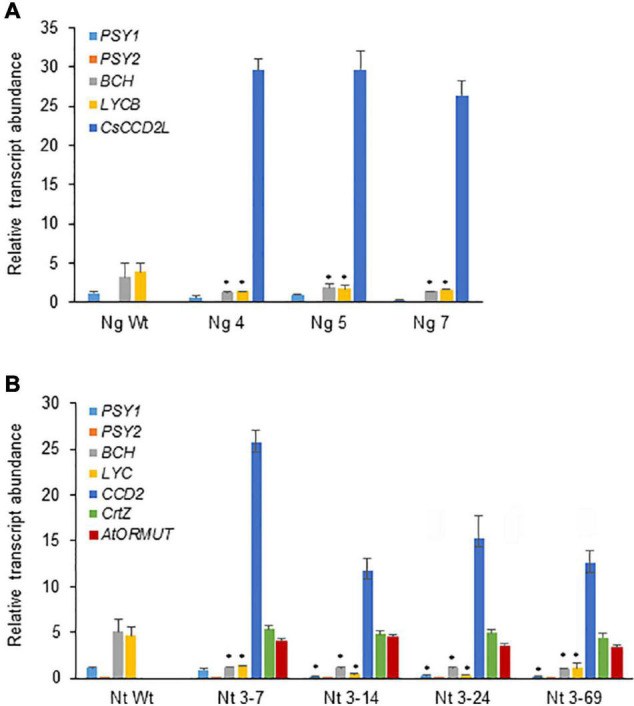
Relative transcript abundance of different transcripts in *Nicotiana glauca* **(A)** and *N. tabacum* **(B)**, wild-type (Wt) and selected transgenic lines for crocin accumulation (Ng lines expressing *CsCCD2L* gene; Nt lines coexpressing *CsCCD2L*, *BrCrtZ*, and *AtOrMut* genes). RT-qPCR was used to quantify gene expression levels in three biological replicates per sample. Error bars represent the SD. The asterisk above the bars indicates *P*-values (Student’s *t*-test) statistically significant (*P* < 0.05).

The expression levels of transgenes and selected endogenous carotenoid biosynthetic genes (*PSY1*, *PSY2*, *LCYB*, and *BCH*) in leaves were monitored by RT-qPCR (Figures 8A,B). The expression of *CsCCD2L* in transgenic *N. tabacum* plants was much higher than those of *BrCrtZ* or *AtOrMut* genes, implying that the activity of the CaMV35S promoter for *CsCCD2L* is much stronger than those of tobacco polyubiquitin Ubi.U4 and the *Arabidopsis* AtUBQ10 promoters for *BrCrtZ* and *AtOrMut* genes, respectively. Endogenous carotenoid biosynthetic genes, including phytoene synthase (*PSY1* and *PSY2*) genes, lycopene β-cyclase (*LCYB*) and β-carotene hydroxylase (*BCH*) genes, were downregulated in all the transgenic plants as compared with the Wt control (Figures 8A,B).

## Discussion

Crocins are mainly known for being responsible for the color of the saffron spice. In addition to their pigment capacity, crocins show bioactive properties with several therapeutic and pharmacological applications (Mykhailenko et al., 2019). The saffron apocarotenoids had a global market size valued at 374.6 million USD in 2020 and is expected to reach USD 721.5 million by 2028, being the medical application segment the one projected to be the fastest-growing segment.^1^ Between 120,000 and 200,000 flowers are needed to produce 1 kg of dried saffron stigma threads, which equates to 370–470 h of work. Consequently, the process is very labor-intensive and risky since it is highly dependent on environmental conditions, leading to high costs.

To overcome these limitations for obtaining saffron apocarotenoids while considering the advances of metabolic engineering, its progression into synthetic biology, and the elucidation of the saffron pathway, we were able to engineer plants from both *N. glauca* and *N. tabacum* expressing *CsCCD2L*. Several efforts have been made by the scientific community to transfer the synthesis of apocarotenoids from saffron to other hosts, looking for the low-cost production of these rare metabolites. It is known that the use of the saffron CsCCD2L enzyme resulted in a maximum accumulation of 1.22, 15.70, and 4.42 mg/l of crocetin in *Saccharomyces cerevisiae* (Chai et al., 2017; Tan et al., 2019) and *Escherichia coli* (Wang et al., 2019). In a subsequent study aimed to confirm, *in planta*, the role of a novel UGTs in picrocrocin biosynthesis (Diretto et al., 2019), *Nicotiana benthamiana* leaves were transiently transformed, *via A. tumefaciens*, with *CsCCD2L*, alone or in combination with *UGT709G1*, which led to the production of 30.5 μg/g DW of crocins, with a glycosylation degree ranging from 1 to 4 (crocin 1–4). In addition, Marti et al. (2020), using a virus-driven expression of *CsCCD2L* in adult *N. benthamiana* plants demonstrated that *CsCCD2L* expression alone is sufficient for significant crocin accumulation in transiently transformed tobacco (up to 2.18 mg/g DW) (Marti et al., 2020).

The expression of *CsCCD2L* in *N. glauca* and *N. tabacum* allowed the accumulation of notable amounts of up to 400 and 36 μg/g DW of crocins, respectively. The underlying reason for the accumulated crocin variation observed among the analyzed *N. glauca* and *N. tabacum* lines could be due to the higher transcript levels of *CsCCD2L N. glauca* lines, and as well to the higher levels of zeaxanthin present in the leaves of *N. glauca* plants. The amount of crocins obtained is much higher in lines from *N. glauca* than in those from *N. tabacum* and the ones previously described using an *A. tumefaciens*-mediated transient expression of the *CsCCD2L* in *N. benthamiana* leaves, alone or in combination with *UGT709G1* (30.5 μg/g DW of crocins). However, this accumulation is lower than the one reported in leaves of adult *N. benthamiana* using a recombinant virus that expressed *CsCCD2L* (2.18 mg/g DW of crocins) (Marti et al., 2020). However, crocin production in *N. gluaca* provides be more effective compared to the data obtained for the production of other carotenoids in this plant; expression of the β-carotene ketolase (*CrtO*) gene led to a total ketocarotenoid concentration in leaves of 136.6 (young) or 156.1 (older) μg/g dry weight and in petals of 165 μg/g dry weight (Zhu et al., 2007; Mortimer et al., 2017).

The profiles of crocins were different from those reported in saffron stigma, where *trans*-crocin 4, followed by *trans*-crocin 3 were the major crocins detected. In contrast, in our transgenic plants, *trans*-crocin-3 was more abundant than *trans*-crocin-4. In *N. benthamiana* leaves expressing transiently only *CsCCD2L*, the major crocin species were the *trans*-crocin 3, and *trans*-crocin with two glucose molecules (Supplementary Figure 3). These data indicated that different endogenous UGTs belonging to *Nicotiana* species recognize crocetin and crocins and can transfer different glucose molecules by changing the qualitative pattern. However, other factors, such as the presence of hydrolase activities that affect the stability of the different crocins synthesized in tobacco cells, and the transport of these crocins to the vacuole and their stability therein, could be influencing the different profiles observed.

It is well-known that *BCH* overexpression resulted in increased zeaxanthin and xanthophyll contents, both in microbes and plants (Lagarde et al., 2000; Du et al., 2010; Arango et al., 2014). Therefore, overexpression of *BCH* could enhance the levels of zeaxanthin as the preferred substrate of CsCCD2L (Frusciante et al., 2014). On the other hand, the *Orange* gene has proven to play an important role in carotenoid accumulation by activating chromoplast differentiation in non-green tissues (Lu et al., 2006), although its expression in green tissues exerts no effect on carotenoid levels (Yuan et al., 2015), we decided to determine whether its introduction could increase the carotenoid content by promoting the formation of carotenoid-sequestering structures. In this context, *BrCrtZ* and *AtOrMut* were introduced in *N. tabacum* to boost the crocin content, since the accumulation of these metabolites was 10-fold lower than *N. glauca*. The introduction of the two transgenes together with *CsCCD2L* has most likely led to an increase in the carotenoid pool used as a substrate by the enzyme and, consequently, in a 3.5-fold increase in crocin compared to the sole expression of *CsCCD2L*.

By introducing *CsCCD2L* alone or in combination with *BrCrtZ* and *AtOrMut*, a decreased level of the major and other minor carotenoids normally present in *N. glauca* and *N. tabacum* was detected. Among all the carotenoids detected, lutein was one of the carotenoids showing the higher decrease, likely due to the ability of *CsCCD2L* to act over the β-ring of this molecule as reported in previous *in vitro* studies where lutein can act as a substrate of this cleavage activity (Frusciante et al., 2014). Zeaxanthin, the crocin precursor, is not a major carotenoid in *Nicotiana* plant leaves, although the production of the crocins in *N. glauca* and *N. tabacum* suggests that the expression of *CsCCD2L* can drag the leaf metabolic flux toward the production of these apocarotenoids. The level of β-carotene was also lower among the *N. glauca* transgenic plants, the same phenomenon has been shown in *N. tabacum* plants transformed with the three genes suggesting a hydroxylation of β-carotene to zeaxanthin under the influence of *CsCCD2L* in *N. glauca* and the combination of the exogenous *BrCrtZ* and *CsCCD2L* in *N. tabacum*. Gotz et al. (2002), previously described the increase of zeaxanthin in tobacco plants (*N. tabacum* L. cv. Samsun) by transformation with a heterologous carotenoid gene encoding β-carotene hydroxylase (CrtZ) from *Erwinia uredovora* under constitutive promoter control. The increase of zeaxanthin in transgenic plants is due to the catalytic activity of the additional β-carotene hydroxylase rather than being primarily caused by an elevation in the conversion of violaxanthin to zeaxanthin in high light (Gotz et al., 2002).

It has been reported that the expression of an appropriate *CsCCD2L* in *N. benthamiana* is sufficient to activate the apocarotenoid pathway in this plant (Diretto et al., 2019; Marti et al., 2020). It seems that both *N. glauca* and *N. tabacum* followed the same pattern as *N. benthamiana*. The crocins produced in this report, using metabolically engineered *N. glauca* (400 μg/g DW) and *N. tabacum* (136 μg/g DW), are lower than those obtained in *N. benthamiana* (2 mg/g DW) using a virus-driven system expressing transient *CsCCD2L*. However, the stable expression of *CsCCD2L* can compensate for the low apocarotenoid yields leading to higher apocarotenoid productivities by reducing costs and labor. Further increase of *N. glauca* and *N. tabacum* content in these apocarotenoids can also be achieved by combining different types of promoters and the expression of other carotenogenic genes to increase the content of the precursors of crocins, zeaxanthin, and lutein. Thus, an approach of focused metabolic engineering intended toward the production of crocins will establish *N. glauca* as a potential industrial host for the production of these apocarotenoids. *N. glauca* is not only a better system for crocin biosynthesis and accumulation than *N. tabacum* but also a nicotine-free species and perennial plant from which the leafy biomass can be harvested repeatedly for crocin production.

## Data Availability Statement

The original contributions presented in the study are included in the article/Supplementary Material, further inquiries can be directed to the corresponding author/s.

## Author Contributions

CZ, OA, PC, and LG-G conceived, designed the research, and wrote the manuscript. CZ, OA, XH, AR-M, TC, and LG-G conducted the experiments. CZ, OA, XH, AR-M, TC, PC, and LG-G analyzed the data. All authors read and approved the final version of the manuscript.

## Conflict of Interest

The authors declare that the research was conducted in the absence of any commercial or financial relationships that could be construed as a potential conflict of interest.

## Publisher’s Note

All claims expressed in this article are solely those of the authors and do not necessarily represent those of their affiliated organizations, or those of the publisher, the editors and the reviewers. Any product that may be evaluated in this article, or claim that may be made by its manufacturer, is not guaranteed or endorsed by the publisher.

## Footnotes

1
https://www.grandviewresearch.com
