# Supplementary material for: Metabolic Engineering of Crocin Biosynthesis in Nicotiana Species
Source: Front Plant Sci. 2022 Mar 8;13:861140. doi: 10.3389/fpls.2022.861140 (PMC8957871; doi:10.3389/fpls.2022.861140)
Supplement: Supplementary file 1 [file Data_Sheet_1.pdf]

Supplemental Table 1. Oligonucleotides used for gene expression analyses and for *CsCCD2L*, *BrCrtZ* and *AtOrMut* gene domestication.

Gene	Forward primer (5´- 3´)	Reverse primer (5´- 3´)	Reference
CsCCD2L	ACATGTCGCCTTGAGAGTCC	TCAGATTTGATGCCAGGTTG	This work
BrCrtZ	TGGCTTACTTGGATCGCTCT	TCCAACAGCAACCATAACGA	This work
AtOrMut	GTTGGTGTGATTTCGGCTTT	GTTTTGGGCGGTGATAGAGA	This work
PSY1	GAAGCCGGAGATCCCTCTCC	TTGCCCAAATAGCCCTTCTTC	(Wang et al., 2021)
PSY2	GGCAGCTGAGATCTACCGATG	TGCACATACTTCGCCACAACG	(Wang et al., 2021)
LCYB	GACAATACAACTAAAGATCTTGATAG	CATAAGCTACTTGATATCCAGGAT	(Wang et al., 2021)
BCH	ATGGCCGCCAGCAGAATTTC	CTCAATTTTCATTTCAATCTCCTCTGTC	(Shi et al., 2014)
Actin	GTATGTCGCCATTCAAGCCGTTCT	ACGGAGGATAGCATGTGGCAAAGCAT	(Moreno et al., 2016)
BrCrtZ domestication	GCGCCGTCTCGCTCGAATGGCTTCTATGATATCCTCTT C	GCGCCGTCTCGCTCAAAGCTCAAGCTCCGCTAGAAGAAG	This work
AtOrMut domestication	GCGCCGTCTCGCTCGAATGTCATCTTTGGGTAGGATTT TG	GCGCCGTCTCGCTCAAAGCTCAATCGAAAGGGTCGATAC G	This work
CsCCD2L-1 domestication	GCGCCGTCTCGCTCGAATGGAATCTCCTGCTACTAAAT TA	GCGCCGTCTCGTTGTCTCTGCCTCCTCCTTA	This work
CsCCD2L-2 domestication	GCGCCGTCTCGACAAGTAAGAAGAAGCCCAAAC	GCGCCGTCTCGCTCAAAGCTCATGTCTCTGCTTGGTGCT	This work

Supplemental Table 2. Carotenoid content (µg/g DW) of leaf tissues of *Nicotiana tabacum* wild type (WT) and transformants co-expressing *CsCCD2L*, *BrCrtZ* and *AtOrMut* genes.

Carotenoids	Nt WT	Nt 7	Nt 14	Nt 24	Nt 69
Neoxanthin	172.4±12.5	63.60±4.10	56.30±4.80	52.68±3.99	46.10±3.90
Violaxanthin	22.9±2.90	15.02±1.06	14.23±1.23	9.77±0.90	17.50±1.70
Antheroxanthin	84.34±9.21	18.80±2.65	36.90±2.90	13.60±1.41	23.10±2.10
Lutein	1811.77±112.23	312.16±22.66	397.60±38.20	293.21±25.25	294.11±25.22
Zeaxanthin	0	30.25±3.48	29.35±3.40	35.21±6.02	32.97±3.02
Trans-β-carotene	1528.43±130.01	891.50±61.40	907.30±71.60	731.42±33.02	895.70±75.60
Cis-β-carotene	336.05±34.22	197.58±19.58	186.90±17.10	160.74±15.87	195.61±13.45
Lycopene	26,3±1.28	16.80±1.70	19.90±1.50	18.80±1.78	19.70±1.80
Total carotenoids	3955.89	1545.71	1648.48	1315.43	1524.79

Supplemental Table 3. Carotenoid content (μg/g DW) of leaf tissues of *Nicotiana glauca* wild type (WT) and transformants expressing *CsCCD2L* gene.

**Carotenoids**	**Ng WT**	**Ng 4**	**Ng 5**	**Ng 7**
Neoxanthin	187.80±17.93	7.68±0.71	5.45±0.56	8.17±0.70
Violaxanthin	173.24±18.31	13.11±1.11	4.31±0.41	6.20±0.72
Antheroxanthin	88.23±8.28	3.42±0.72	1.35±0.40	1.53±0.58
Lutein	1564.23±160.02	275.33±25.33	159.15±15.05	157.56±17.35
Zeaxanthin	0	101.23±7.66	42.85±5.82	45.08±4.37
Trans-β-carotene	1335.11±129.18	527.84±31.04	221.65±20.69	363.32±23.32
Cis-β-carotene	291.33±20.27	118.01±12.01	39.55±2.55	81.97±6.97
Lycopene	17.9±1.34	11.62±1.74	18.25±1.31	12.13±1.15
Total carotenoids	3639.94	1058.24	492.56	675.96
