# Supplementary material for: Metabolic Engineering of Crocin Biosynthesis in Nicotiana Species
Source: Front Plant Sci. 2022 Mar 8;13:861140. doi: 10.3389/fpls.2022.861140 (PMC8957871; doi:10.3389/fpls.2022.861140)
Supplement: Supplementary file 2 [file Data_Sheet_2.PDF]

A

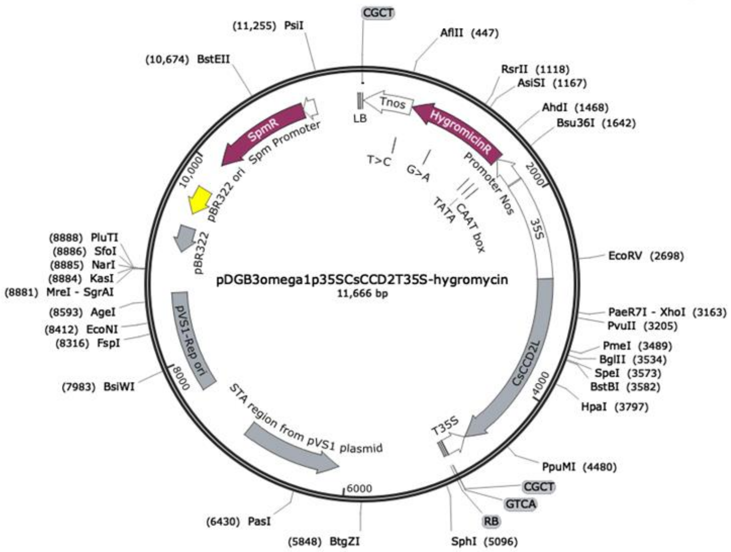

B

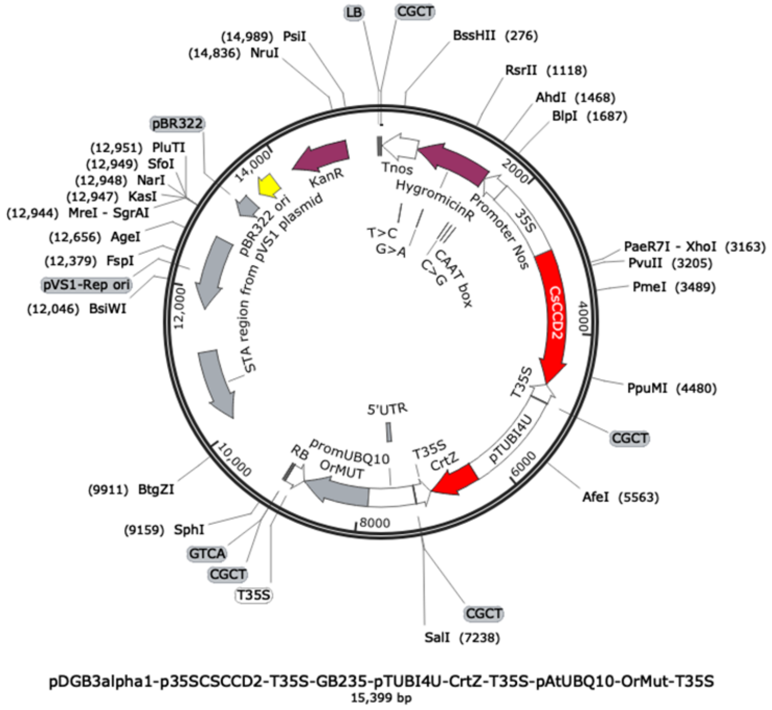

Supplemental Figure 1. Schematic representation of the constructs used to transform *N. glauca* (**A**) and *N. tabacum* (**A** and **B**) plants.

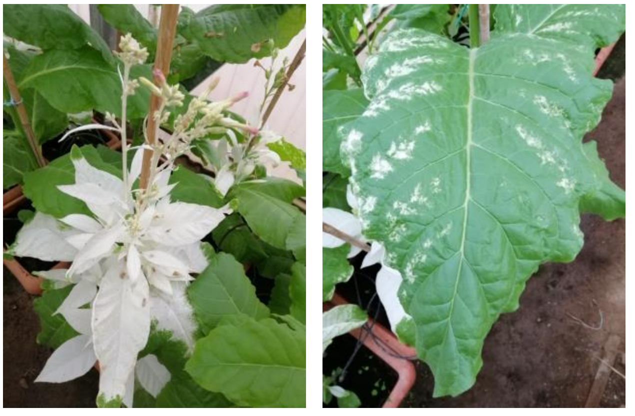

Supplemental Figure 2. Bleaching phenotype of some T0 *N. tabacum* plants overexpressing *CsCCD2L*.

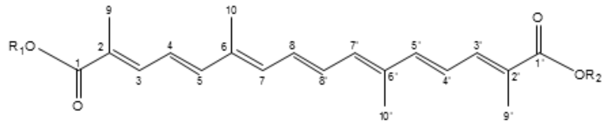

R1=R2=H Crocetin
R1=R2=GG, Crocin-4GG

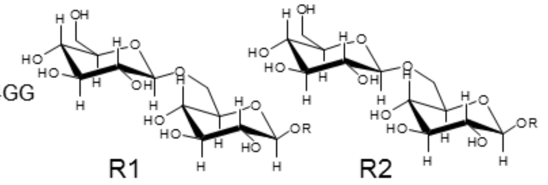

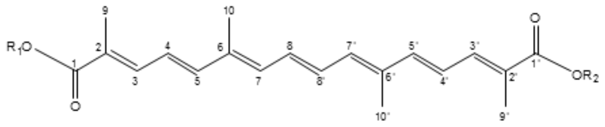

R1=R2=H Crocetin
R1=g R2 =GG Crocin-3Gg

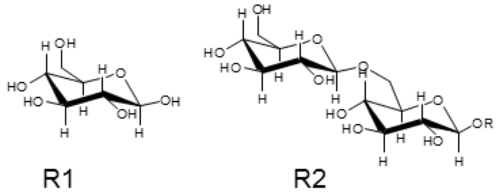

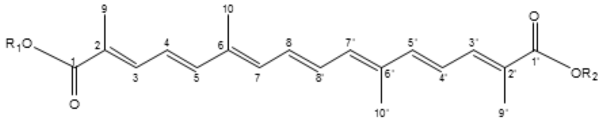

R1=R2=H Crocetin
R1=H R2=GG, Crocin-2G

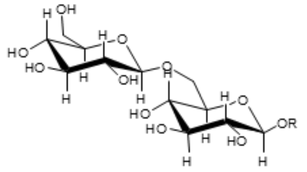

Supplemental Figure 3. Crocins structure
